# High Activity of N-Acetylcysteine in Combination with Beta-Lactams against Carbapenem-Resistant *Klebsiella pneumoniae* and *Acinetobacter baumannii*

**DOI:** 10.3390/antibiotics11020225

**Published:** 2022-02-10

**Authors:** Massimiliano De Angelis, Maria T. Mascellino, Maria C. Miele, Dania Al Ismail, Marisa Colone, Annarita Stringaro, Vincenzo Vullo, Mario Venditti, Claudio M. Mastroianni, Alessandra Oliva

**Affiliations:** 1 Department of Public Health and Infectious Diseases, Sapienza University of Rome, 00185 Rome, Italy; massimiliano.deangelis@uniroma1.it (M.D.A.); mariateresa.mascellino@uniroma1.it (M.T.M.); mariaclaudia.miele@uniroma1.it (M.C.M.); dania.alismail@uniroma1.it (D.A.I.); vincenzo.vullo@uniroma1.it (V.V.); mario.venditti@uniroma1.it (M.V.); claudio.mastroianni@uniroma1.it (C.M.M.); 2 National Center for Drug Research and Evaluation, Italian National Institute of Health, 00161 Rome, Italy; marisa.colone@iss.it (M.C.); annarita.stringaro@iss.it (A.S.)

**Keywords:** N-acetylcysteine, carbapenem-resistant *Klebsiella pneumoniae*, carbapenem-resistant *Acinetobacter baumannii*, multidrug resistance, synergism

## Abstract

Aim: The aim of the study was to evaluate the in vitro activity of N-acetylcysteine (NAC), alone or in combination with beta-lactams, against carbapenem-resistant *Klebsiella pneumoniae* (CR-Kp) and *Acinetobacter baumannii* (CR-Ab). Methods: The antibacterial activity of each compound was tested by broth microdilution and the synergism was evaluated by the checkerboard method. Killing studies of NAC alone and in combination with beta-lactams were performed. Bacterial morphological changes were investigated with scanning electron microscopy (SEM). Results: Overall, 30 strains were included (15 CR-Kp and 15 CR-Ab). The NAC Minimal Inhibitory Concentrations (MIC)50/90 were 5/5 and 2.5/5 mg/mL for CR-Kp and CR-Ab, respectively. For both microorganisms, NAC, in addition to beta-lactams (meropenem for CR-Kp, meropenem and ampicillin/sulbactam for CR-Ab, respectively), was able to enhance their activity. The killing studies showed a rapid and concentration-dependent activity of NAC alone; the addition of NAC to meropenem or ampicillin/sulbactam at subinhibitory concentrations induced a fast and lasting bactericidal activity that persisted over time. The SEM analyses showed evident morphological alterations of the bacterial cells following incubation with NAC, alone and in combination with meropenem. Conclusions: NAC demonstrated a high in vitro activity against CR-Kp and CR-Ab and was able to enhance beta-lactams’ susceptibility in the tested strains. The preliminary data on the SEM analyses confirmed the in vitro results.

## 1. Introduction

Bacterial antibiotic resistance is responsible for high numbers of infections and deaths, and almost 70% of this disease burden is caused by multidrug-resistant Gram-negative bacteria (MDR-GNB) [1]. Indeed, in 2017, the World Health Organization included MDR-GNB as critical priorities for research and drug development [1].

Infections caused by MDR-GNB are generally associated with a poor prognosis and an increased fatality rate of over 40%, especially in the presence of septic shock [2,3,4,5].

In the context of increasing antimicrobial resistance and restricted therapeutic options typical of the contemporary era, there is a growing scientific interest on finding possible therapeutic adjuvants able to increase the antibiotic efficacy for MDR-GNB, including carbapenem-resistant *Klebsiella pneumoniae* (CR-Kp) and *Acinetobacter baumannii* (CR-Ab) [6,7,8]. Amongst them, N-acetylcysteine (NAC), a mucolytic agent with antioxidant and anti-inflammatory properties, has aroused interest also for its antibacterial activity [9]. Indeed, several in vitro studies demonstrated a synergistic interaction with antibiotics against MDR-GNB [10,11,12], and, in addition, a recent clinical study reported preliminary evidence that, in patients with septic shock due to CR-Kp or CR-Ab, the 30-day mortality might be significantly lower in those who receive intravenous NAC as opposed to those not receiving NAC [6].

Based on these premises, the aim of the study was to qualitatively and quantitatively analyze the in vitro activity of NAC, alone and in combination with different antibiotics, against a collection of clinically relevant CR-Kp and CR-Ab strains. Furthermore, we evaluated whether the observed in vitro activity against a CR-Kp exhibiting a high level of meropenem and colistin resistance corresponded to bacterial cell morphological changes by means of scanning electron microscopy (SEM) analyses. Finally, we measured the serum bactericidal activity (SBA) against CR-Kp and CR-Ab in a subject who received a treatment course of 2 days with oral NAC for respiratory infection.

## 2. Results

### 2.1. In Vitro Analyses: CR-Kp

The Minimal Inhibitory Concentrations (MIC)50/90 (μg/mL) were the followings: meropenem (MEM) 256/1024, colistin (COL) 256/1024, rifampin (RIF) 32/256 and tigecycline (TIG) 0.5/1, whereas the MIC50/90 of NAC was 5/5 mg/mL. The combination MEM + NAC showed full synergism in all the tested strains, and the combination RIF + NAC was synergic in 3/15 (20%) strains; on the other hand, no synergism was found when considering TIG + NAC or COL + NAC (Table 1).

**Figure 1:**
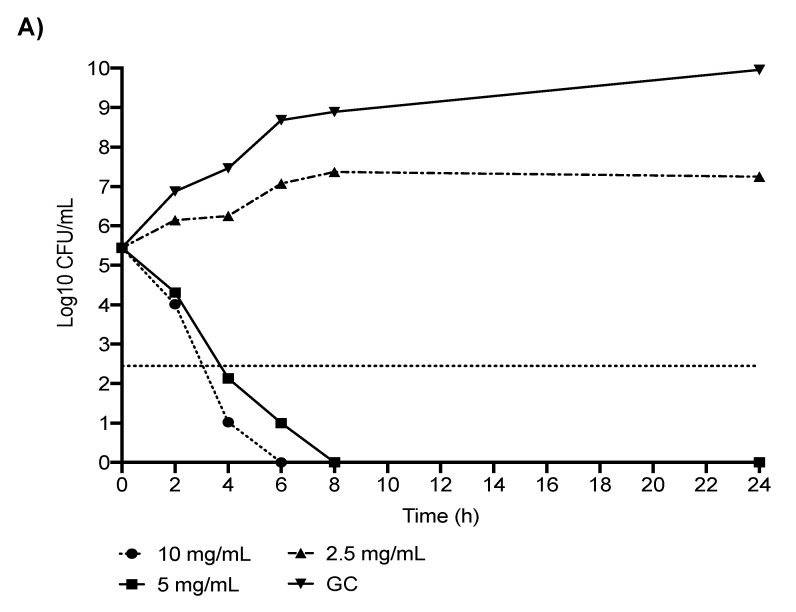

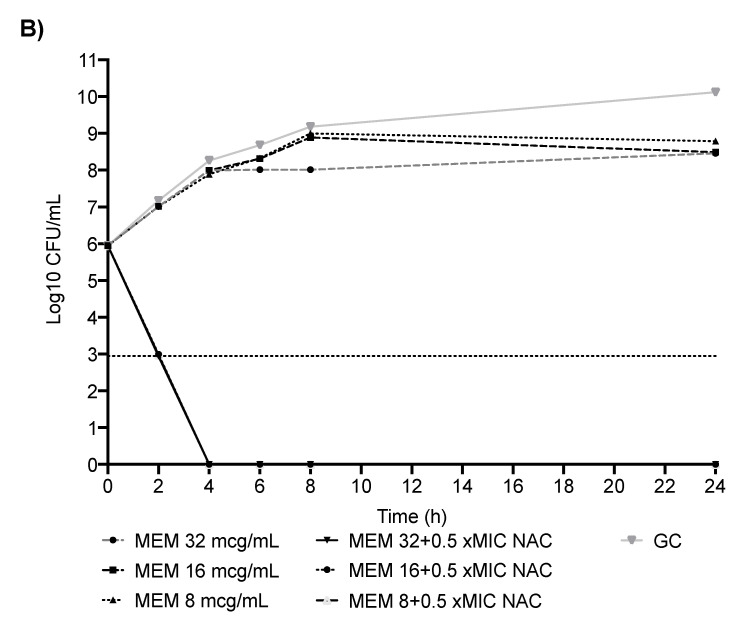
Killing studies of NAC alone (**A**) and in combination with subinhibitory concentrations of MEM (**B**) against carbapenem-resistant *Klebsiella pneumoniae* (MEM MIC 1024 µg/mL and NAC MIC 5 mg/mL). MEM: meropenem; NAC: N-acetylcysteine; GC: growth control. Horizontal dashed line represents the bactericidal activity (≥99.9%, ≥3-log10 CFU/mL reduction of the initial bacterial count at each time point). Values correspond to the mean.

According to the observed high synergistic activity of the MEM + NAC combination, and taking into consideration the very high MEM MICs, which are hardly achievable in the serum, we performed a quantitative analysis by using time killing curves of NAC alone and in association with MEM against a CR-Kp strain exhibiting high level of carbapenem and colistin resistance (1024 µg/mL and 2048 µg/mL, respectively). As observed in Figure 1, when considered alone, NAC exhibited a concentration-dependent bactericidal activity, with a rapid (4 h) bactericidal activity at concentrations of 10 mg/mL (2 × MIC) and 5 mg/mL (1 × MIC) and the absence of bacterial growth at 8 h, which was maintained up to 24 h (Figure 1A). Interestingly, the addition of subinhibitory NAC (2.5 mg/mL, 0.5 × MIC) to MEM at concentrations 32, 16 and 8 µg/mL, which, alone, did not have an effect towards CR-Kp, induced an early and long-lasting bactericidal activity, with an absence of bacterial growth from 4 h of incubation (Figure 1B).

### 2.2. In Vitro Analyses: CR-Ab

Amongst the tested strains, the MIC50/90 (μg/mL) were the followings: MEM 128/512, ampicillin/sulbactam (A/S) 64/256, COL 0.25/4, RIF 2/8 and TIG 0.5/1. NAC MIC50/90 was 2.5/5 mg/mL. Synergism was observed in all NAC + MEM and NAC + A/S combinations and in 14 out of 15 (93.3%) strains for NAC + RIF; in contrast, NAC + COL and NAC + TIG did not show synergistic activity (Table 2).

**Figure 2:**
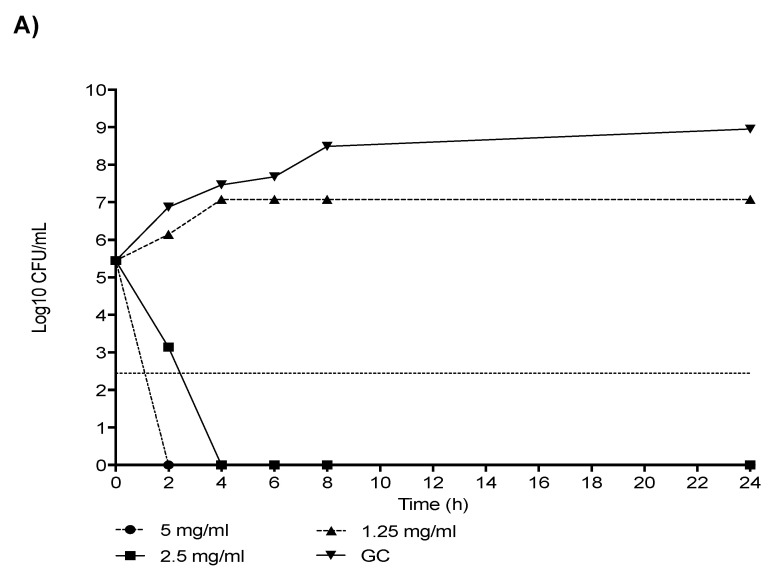

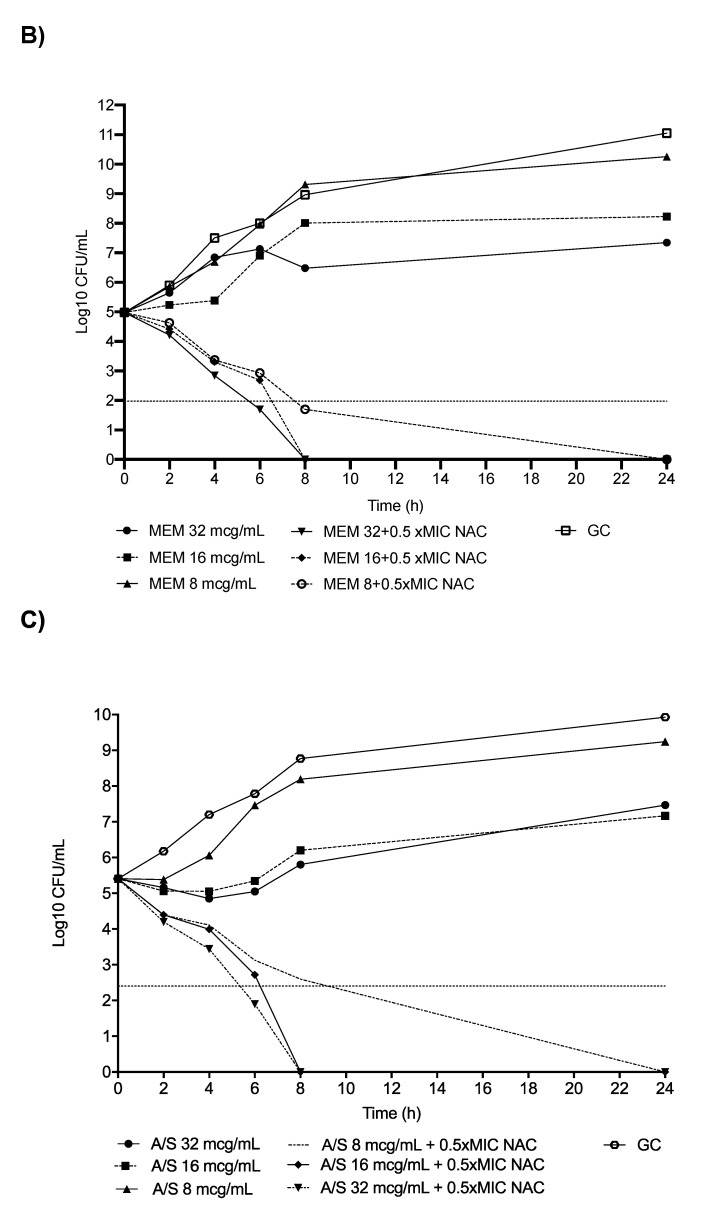
Killing studies of NAC alone (**A**) and in combination with subinhibitory concentrations of MEM (**B**) and A/S (**C**) against carbapenem-resistant *Acinetobacter baumannii* (MEM MIC 1024 µg/mL and NAC MIC 2.5 mg/mL). MEM: meropenem; A/S: ampicillin/sulbactam; NAC: N-acetylcysteine; GC: growth control. Horizontal dashed line represents bactericidal activity (≥99.9%, ≥3-log10 CFU/mL reduction of the initial bacterial count at each time point). Values correspond to the mean.

Similar to what was observed with the CR-Kp strains, the killing studies performed on a representative strain exhibiting a high level of MEM resistance showed a concentration-dependent activity of NAC alone, with rapid bactericidal activity (2 h and 4 h) at 2 × MIC (5 mg/mL) and 1 × MIC (2.5 mg/mL) concentrations, respectively, and afterwards, the absence of bacterial growth up to 24 h (Figure 2A).

Of note, the addition of NAC to subinhibitory and not effective MEM and A/S concentrations (32, 16 and 8 μg/mL for both MEM and A/S) was able to enhance the activity of these antibiotics, being bactericidal at 8 h (Figure 2B,C).

### 2.3. Scanning Electron Microscopy Analyses

**Figure 3:**
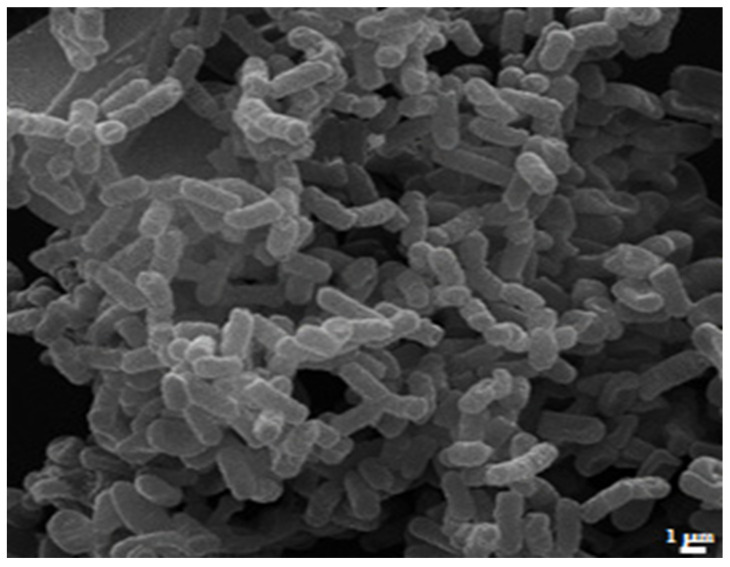
Scanning electron micrographs of untreated (control) resistant cells of *K. pneumoniae*. The typical rod shape of the microorganism and the production of an extracellular matrix can be noted.

**Figure 4:**
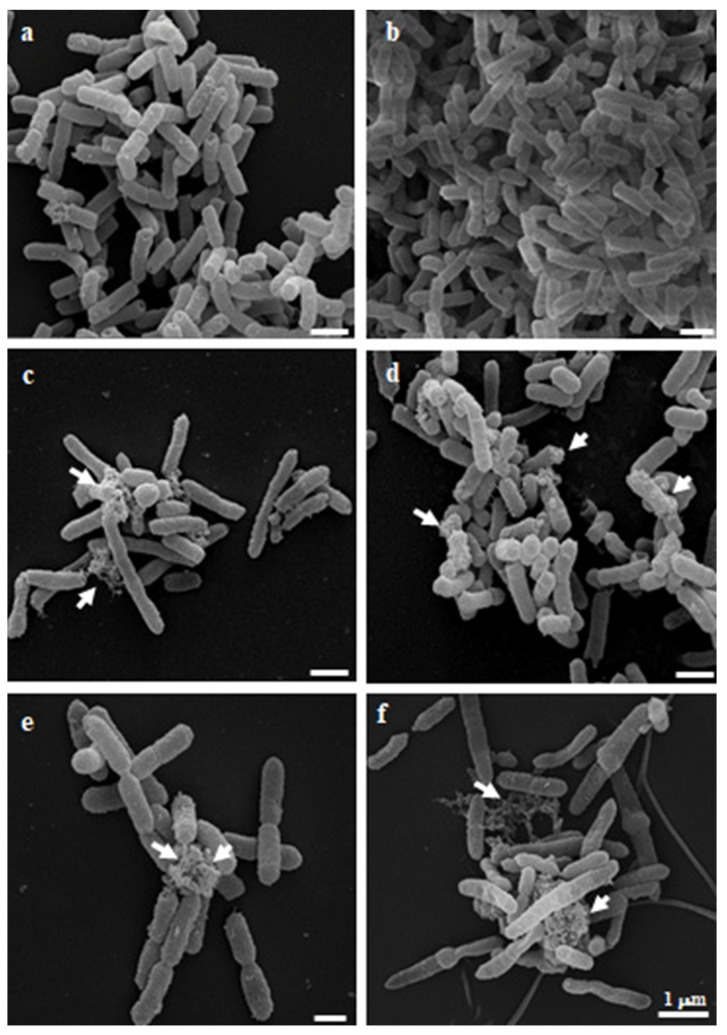
Scanning electron micrographs of resistant *K. pneumoniae* in the presence of MEM alone (**a**,**b**), NAC alone (**c**,**d**) and MEM + NAC (**e**,**f**). (**a**,**b**) Cells treated with MEM 1 and 2 µg/mL, respectively. (**c**,**d**) Cells treated with NAC at subinhibitory concentrations of 2.5 (0.5 × MIC) and 3 mg/mL (0.6 × MIC), respectively. (**e**,**f**) Cells treated with the combinations of MEM 1 µg/mL + NAC 0.5 × MIC (2.5 mg/mL) and MEM 2 µg/mL + NAC 0.5 × MIC (2.5 mg/mL), respectively. Arrows indicate outer membrane vesicles (OMVs).

Given the availability of a CR-Kp strain exhibiting high MEM and COL MICs, we performed SEM analyses to evaluate whether the observed in vitro antibacterial activity of NAC, alone and in combination with MEM, corresponded to morphological alterations of the bacterial cells. Figure 3 shows the appearance of CR-Kp in the absence of antibiotic treatment (Control), in which the typical rod shape of the microorganism and the production of an extracellular matrix can be noted. In Figure 4a,b, the morphology of the bacteria in the presence of subinhibitory concentrations of MEM (1 and 2 µg/mL, respectively) are presented. Again, no difference in the appearance and morphology of the bacterium compared to the control could be appreciated, thus confirming that MEM alone is not effective. Similar to what observed in the in vitro experiments, the effect of NAC at subinhibitory concentrations (2.5 (0.5 × MIC) and 3 (0.6 × MIC) mg/mL, respectively) on the morphology of the tested bacteria was evident at SEM images (Figure 4c,d respectively) that showed bacterial elongation and the presence of outer membrane vesicles (OMVs, arrows), possibly in a concentration-dependent manner.

Interestingly, the combinations MEM 1 µg/mL + NAC 0.5 × MIC (2.5 mg/mL) (Figure 4e) and MEM 2 µg/mL + NAC 0.5 × MIC (2.5 mg/mL) (Figure 4f) showed similar, although more pronounced, patterns of bacterial cell integrity perturbations. The figure shows bacterial elongation, especially after the MEM 2 µg/mL + NAC 0.5 × MIC (2.5 mg/mL) treatment. The cells were merged with many damages, as if the outer cell wall and inner membrane would break down. In addition, a reduction in the number of bacteria could be observed.

### 2.4. Serum Bactericidal Activity

**Figure 5:**
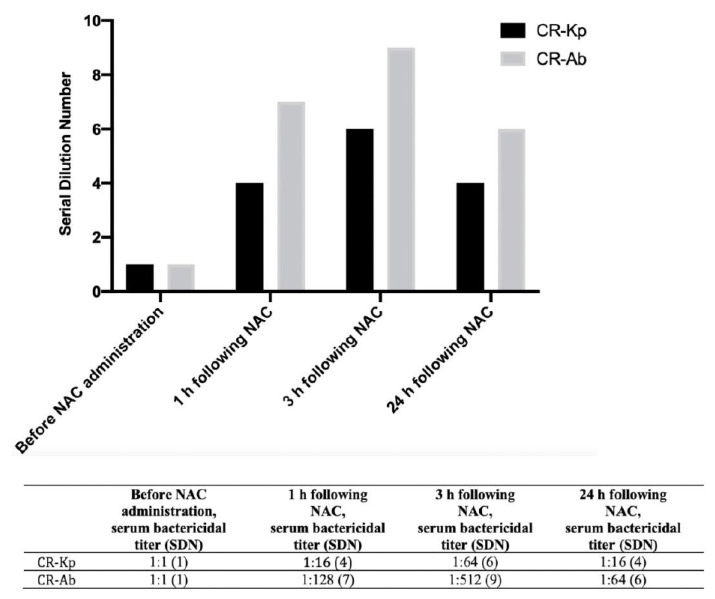
Serum bactericidal activity (expressed by the serial dilution number (SDN, upper panel) and by the serum bactericidal titers (lower panel)) against representative carbapenem-resistant (CR) *K. pneumoniae* and CR *A. baumannii* before and after the assumption of oral (600 mg) NAC administration. The serial dilution number corresponded to the number of the serial 2-fold serum dilutions needed to obtain the corresponding serum bactericidal titer. CR-Kp: carbapenem-resistant *K. pneumoniae*; CR-Ab: carbapenem-resistant *A. baumannii*; NAC: N-acetyl-cysteine; SDN: serial dilution number.

Before NAC administration, the serum did not have antibacterial activity towards CR-Kp, whereas after 1 h and 3 h following NAC administration, the SBA was 1:16 and 1:64, respectively, which was maintained, albeit reduced, up to 24 h (1:16). With regard to CR-Ab, the SBAs were 1:1, 1:128 and 1:512 before, after 1 h and after 3 h of NAC administration, respectively, with a reduced activity at 24 h (1:64) (Figure 5). The corresponding serum dilution numbers (SDN) are shown in Figure 5.

## 3. Discussion

NAC, the N-acetyl derivative of the amino acid L-cysteine, is a mucolytic agent also exhibiting antioxidant and anti-inflammatory properties thanks to the increase of glutathione able to reduce free oxygen radicals and to inhibit the effect of proinflammatory cytokines [9,13]. Currently, NAC is mostly used for the management of lower respiratory tract conditions characterized by high thick mucus production (for instance, patients with cystic fibrosis or Chronic Obstructive Pulmonary Disease) [12]; nevertheless, since NAC also possesses antibacterial activities, a renewed interest regarding its use in the field of infections has mounted in the recent years, and therefore, a potential role as adjuvant to traditional antimicrobial therapy has been suggested [6,10,11].

In the present study, we demonstrated that (i) NAC alone exhibited potent antibacterial activity against CR-Kp and CR-Ab in a concentration-dependent manner, (ii) NAC was highly synergic with both meropenem and ampicillin/sulbactam by restoring their susceptibility and (iii) NAC’s antibacterial activity correlated with the bacterial morphological changes, as shown by the SEM analysis.

So far, several studies have shown in vitro antibacterial and antibiofilm activity of NAC against both Gram-positive and Gram-negative microorganisms, including, amongst others, relevant difficult-to-treat pathogens such as *P. aeruginosa*, *S. maltophilia* and the *Burkholderia cepacia* complex [12,14,15,16,17,18,19]. More recently, Pollini et al. showed a potent synergism between NAC and colistin against colistin-resistant CR-Ab, whereas no activity was observed when the strains were susceptible to colistin. Furthermore, a remarkable antibiofilm activity of NAC in combination with colistin was shown [10]. The same research group found similar results when studying the combination NAC plus colistin against planktonic and biofilm *S. maltophilia*, again in a dose-dependent manner [11]. Taken these data together, the authors suggested that the topical administration of NAC in addition to inhaled colistin may contribute not only to potentiate colistin antibacterial activity but also to revert colistin resistance phenotype and exert antibiofilm activity [10,11]. On the other hand, controversial results were obtained when evaluating the activity of NAC in combination with beta-lactams. Indeed, Goswami et al., who investigated the in vitro activity of NAC against *K. pneumoniae*, *E. coli* and *P. aeruginosa*, found that, in the presence of NAC, the ampicillin activity was remarkably augmented in most of the strains and therefore concluded that, although NAC could have had a negative effect on the aminoglycoside or fluoroquinolone antibacterial activity, it was able to enhance the efficacy of beta-lactams against several bacterial strains [20]. Conversely, Moon and colleagues found that the antibiotics susceptibility of planktonic *Prevotella intermedia* was not affected by NAC and that the activity of ampicillin decreased in the presence of NAC [21]. Likewise, Rodriguez-Bertram et al. showed that not only did NAC not potentiate different antibiotic activity against *P. aeruginosa*, but interestingly, the strains initially susceptible to imipenem became imipenem-resistant in the simultaneous presence of NAC and imipenem, possibly by a competitive inhibition of imipenem uptake through OprD exerted by NAC; on the other hand, the activity of meropenem was only slightly affected by NAC [22]. Notably, the authors found that the observed antimicrobial effect of NAC depended more on the NAC solution’s pH rather than on the NAC activity itself [22]. Landini et al. investigated the effect of NAC alone and in combination with different beta-lactams against several Gram-negative and Gram-positive microorganisms, including one strain of KPC-producing *K. pneumoniae*, and found that NAC MICs exceeded 16 mg/mL in most strains. In particular, no synergistic interaction was observed following the NAC addition to antibiotics. Finally, the authors also showed that carbapenem activity was negatively influenced by NAC, with, again, imipenem more affected than meropenem and ertapenem [23].

Differently from what has been previously reported, in the present study, no synergism was found when the combination NAC plus colistin was tested against both CR-Kp and CR-Ab, and this result was independent from colistin susceptibility or resistance; on the other hand, a remarkable synergism was observed when combining NAC with meropenem and ampicillin/sulbactam. Of note, these findings did not depend on low pH values of NAC solution, since pH was checked daily before performing each experiment and it was always within 7.0–7.2 values. Furthermore, among carbapenems, we tested meropenem and not imipenem, since it has been demonstrated to be less influenced by NAC and more stable in solution than imipenem [23]. Finally, the detrimental effect of NAC on carbapenems was mostly evident at high NAC concentrations (≈8.16 mg/mL), greater than those used in our study. As a matter of fact, in the present study, we used subinhibitory NAC concentrations (1.25–2.5 mg/mL), which reflected the levels achievable in the case of a topical administration of NAC [23]. On the other hand, these concentrations were lower than the serum drug concentrations following intravenous NAC administration [24], suggesting that the adjuvant action of NAC may be present even at very low systemic concentrations with reduced side effects. These in vitro findings are in line with the very preliminary evidence of the potential benefit on mortality by adding intravenous NAC to systemic antibiotics in septic shock due to CR-Kp and CR-Ab [6].

To corroborate the above-described in vitro results, we performed SEM analyses on a representative highly carbapenem and colistin-resistant CR-Kp strain, and we found that, in the presence of low meropenem concentrations, no alterations could have been observed in the bacterial shape and in its structure. Conversely, when the strain was challenged with NAC alone or with NAC in combination with low meropenem concentrations, a pronounced alteration of the bacterial structure was found, with bacterial elongation, perturbation of the cell integrity, outer cell wall or inner membrane breakdown and OMVs. Interestingly, among the numerous favorable meanings that are attributed to the presence of OMVs in bacteria (for instance, mediating bacterial survival, allowing the maintenance of virulence and playing a role in bacterial adaptation) [25], there is also the hypothesis that toxic substances may be expelled by exocytosis and incorporated in the OMVs, which become visible at the SEM.

**Figure 6:**
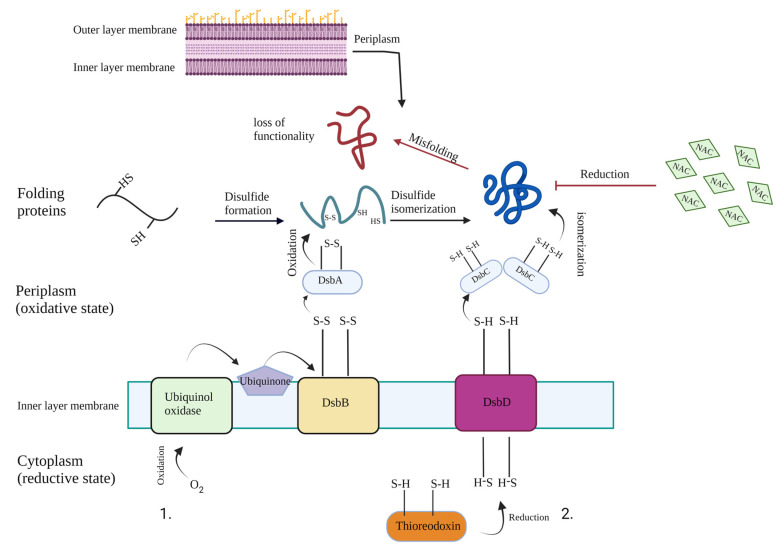
Disulphide Bond Proteins (Dsb) A, B, C and D regulate the oxidation state of Gram-negative bacteria periplasm and contribute to the definite configuration of bacterial proteins. Among the post-translational modifications of periplasmic proteins, there is the formation of disulfide bridges (S–S), which allow protein correct folding and their spatial arrangement. The formation of disulfide bonds is mediated by DsbA and is further isomerized by DsbC to reach the correct proteins configurations. 1 and 2 indicate the flow of electrons in the DsbB–DsbA oxidative pathway and the DsbD–DsbC reductive pathway, respectively. By means of reducing the effect, N-acetyl cysteine is able to inhibit this process and alter the redox equilibrium of the periplasm by breaking the disulphide bridges and, ultimately, rendering the protein inactive (protein misfolding).

Several mechanisms may be hypothesized for the antibacterial activity of NAC [9], and amongst others, a relevant role is exerted by the functional group -SH, which is able to break the S–S disulfide bridges of proteins present in the bacterium, thus denaturing their dimensional structure and, ultimately, inactivating their function. For instance, thiol disulfide oxidoreductases, including Disulphide Bond Protein (Dsb) A, DsbB, DsbC and DsbD, are a set of enzymes that finely control the redox state in the periplasm of Gram-negative bacteria (Figure 6). In fact, the concerted action of these enzymes makes possible the tertiary configuration of the proteins produced by the bacterium, generating an oxidative state in the periplasm that allows the formation of disulfide bridges, thus giving rise to the native conformation of the protein and allowing their specific functionality. Conversely, the presence of a NAC thiol group may alter the redox state of bacterial periplasma, thus inactivating this refined regulated process and generating misfolding of the proteins, which accumulate in the cytoplasm and undergo exocytosis throughout the formation of OMVs, as demonstrated by the SEM analyses [26]. The involvement of DsbA as a main actor in this process was elegantly demonstrated by Paxman and colleagues in 2009 by showing that a nonsense mutation in the *E. coli* DsbA protein was able to inactivate beta-lactamases and, accordingly, enhance the sensitivity to penicillin [27]. Similarly, periplasmic bacterial enzymes such as carbapenemases and other beta-lactamases are subjected to the same refined regulatory system and, in the presence of NAC, are inactivated by means of protein misfolding and, ultimately, lose their function. This explanation of NAC’s antibacterial activity is coherent with the observed structural alterations at the SEM, which, at the end, are responsible for the observed in vitro absence of bacterial growth in the presence of NAC. Given these promising results, further studies are warranted to better explore the interaction between NAC and the Dsb pathway.

The present study has several limitations: first, amongst the currently available therapeutic armamentarium, only the interactions with MEM and A/S were explored in the current study. Nevertheless, we believe that these encouraging findings, albeit still preliminary, may open the path towards additional future investigations regarding the adjuvant in vitro effect of NAC on the new beta-lactam/beta-lactamase inhibitors, such as ceftazidime/avibactam and meropenem/vaborbactam, which we could not test in the present report. Second, SEM analyses were performed only against a CR-Kp strain exhibiting a high level of MEM and COL; anyhow, the coexistence of a high level of MEM and COL resistance, with the latter possibly influencing itself the bacterium permeability or its morphology, has brought us the unique possibility of investigating the effect of NAC on bacterial cell morphology. Lastly, the presence of outer porins in the tested strains was not investigated, and therefore, we could not generalize our results to all the existing carbapenems; anyhow, we believe these results mostly apply to meropenem and still need confirmation for imipenem.

## 4. Materials and Methods

### 4.1. In Vitro Analyses

A total of 30 clinically relevant strains (15 CR-Kp and 15 CR-Ab) isolated from blood, tracheal aspirate, sputum, bronchoalveolar lavage, urine, purulent drainage and rectal swabs were analyzed. Carbapenem resistance, as well as the determination of the type of carbapenemases (when present), were evaluated as for routine practice at the Department of Public Health and Infectious Diseases, Sapienza University of Rome.

Minimal inhibitory concentrations (MIC) were performed by the broth microdilution (BMD) method in accordance with the guidelines [28]. Antimicrobial agents were provided as purified powder by the manufacturer (Merck KGaA, Darmstadt, Germany, formerly known as Sigma Aldrich, Italy). Stock solutions at different concentrations were prepared in sterile and pyrogen-free 0.9% saline or water, according to the manufacturer’s instructions.

The checkerboard method was used to qualitatively investigate the synergism of the following combinations for CR-Kp: NAC + MEM, NAC + COL, NAC + RIF and NAC + TIG, whereas, for CR-Ab, NAC + MEM, NAC + A/S, NAC + RIF, NAC + COL and NAC + TIG were evaluated. Briefly, a 96-well microtiter plate containing NAC/antibiotic combinations at different concentrations was used to perform checkerboard synergy testing. Wells containing a final inoculum of ~5 × 105 CFU/mL were incubated at 37 °C for 24 h under static conditions in Mueller–Hinton Broth (MHB). The fractional inhibitory concentration index (FICI) of each combination was defined as: ∑FIC: FICA + FICB = MICA + B/MICAalone + MICB + A/MICBalone. Synergism was defined as FICI ≤ 0.5.

Given their high synergistic activity, the combinations NAC + MEM and NAC + A/S were also investigated by means of time-killing studies performed in the logarithmic growth phase using an initial inoculum of ~5 × 10^5^ CFU/mL on representative CR-Kp and CR-Ab strains exhibiting a very high level of MEM resistance and NAC MICs of 5 and 2.5 mg/mL, respectively. The killing curves were performed in borosilicate glass tubes in a final volume of 10-mL CAMHB, which were further incubated at 37 °C. At 2, 4, 6, 8 and 24-hour time points, 1-mL aliquots were sampled and washed with 0.9% saline solution in order to prevent the antibiotic carryover effect. Ten-fold dilutions were then plated on Muller–Hinton agar, and the number of CFUs was determined. Medium without antibiotics was used as the growth control. Bactericidal activity was defined as ≥99.9% (i.e., ≥3-log10 CFU/mL) reduction of the initial bacterial count at each time point. Synergy was defined as a ≥100-fold decrease in CFU/mL between the combination and its most active constituent at the same concentration after 24 h, with the number of surviving organisms in the presence of the combination ≥100-fold CFU/mL below the starting inoculum. NAC alone was tested at concentrations 2 × MIC, 1 × MIC and 0.5 × MIC, whereas, in combination with MEM, we used the following concentrations: MEM 32 μg/mL, MEM 16 μg/mL, MEM 8 μg/mL, NAC 0.5 × MIC + MEM 32 μg/mL, NAC 0.5 × MIC + MEM 16 μg/mL and NAC 0.5 × MIC + MEM 8 μg/mL for both CR-Kp and CR-Ab. Likewise, for the NAC + A/S combination, the following concentrations were used: A/S 32 μg/mL, A/S 16 μg/mL, A/S 8 μg/mL, NAC 0.5 × MIC + A/S 32 μg/mL, NAC 0.5 × MIC + A/S 16 μg/mL and NAC 0.5 × MIC + A/S 8 μg/mL. We decided to use these concentrations of MEM, since they reflect the serum achievable drug levels in the case of high-dosage meropenem administration [29]. pH values of the NAC solution were daily checked before performing each experiment, and the pH was maintained always within 7.0–7.2.

All in vitro experiments were performed in duplicate.

### 4.2. Scanning Electron Microscopy Analyses

SEM analyses were performed on a representative CR-Kp strain exhibiting a high level of MEM and COL resistance.

Briefly, bacteria (2.3 colonies) were incubated with NAC (at concentrations of 2.5 mg/mL (0.5 × MIC) and 3 (0.6 × MIC) mg/mL) and/or MEM (at the concentrations of 1 and 2 µg/mL) in tryptic soy broth (TSB) for 24 h at 37 °C in static conditions. Controls were bacterial cells incubated for 24 h at 37 °C in static conditions in TSB.

After incubation, the drug-resistant CR-Kp strains (control and treated cells) were fixed for 1 h at room temperature with 2.5% (*v*/*v*) glutaraldehyde in 0.2-M cacodylate buffer (pH 7.4). After 3 washes in the same buffer, the cells were post-fixed with 1% (*w*/*v*) OsO4 for 1 h. Then, the samples were deposited on 12-mm-diameter glass coverslips for 30 min and dehydrated using an increasing ethanol series of 35%, 50%, 70%, 85%, 96% and 100% replaced by CO_2_ through a critical point dry instrument and, finally, gold coated by sputtering (SCD 040 Balzers device, Bal-Tec). The samples were examined at 30 kV with a Field Emission Gun Scanning Electron Microscope (Inspect F-FEI, USA).

### 4.3. Serum Bactericidal Activity

The Serum Bactericidal Activity (SBA) was performed on a subject who received a 2-day oral NAC (600 mg) for respiratory thick secretions measured at 1 h, 3 h and 24 h from NAC administration. These time points were chosen according to the peak of plasma concentrations after oral administration, which approximately occurred at 2 h [30]. The SBA was tested against the CR-Kp (*n* = 1) and CR-Ab (*n* = 1) strains used in the killing studies, namely representative CR-Kp and CR-Ab strains exhibiting a very high level of MEM resistance and NAC MICs of 5 and 2.5 mg/mL, respectively.

According to the method described by Stratton [31], two-fold serial dilutions of the test sample in human serum were prepared in microtiter plates. For each strain, exponentially growing cultures were diluted to ~10^6^ CFU/mL in Mueller–Hinton broth supplemented with Mg^2+^ and Ca^2+^, and 50 µL of the bacterial suspension was distributed into each well to give a final bacterial concentration of 5 × 10^5^ CFU/mL. Aliquots of 10 µL each were withdrawn from the wells that did not show visible growth and were subcultured on Trypticase Soy Agar supplemented with blood. A colony count was performed after 48 h of incubation at 35 °C. SBA was defined as the highest dilution of each sample that reduced the initial inoculum by ≥99.9% [32].

The SBA was reported as a serum bactericidal titer and as a serial dilution number (SDN), which, in accordance with previous reports [33], corresponded to the number of the serial two-fold serum dilutions needed to obtain the corresponding serum bactericidal titer.

The subject receiving NAC gave informed written consent for the determination of SBA.

## 5. Conclusions

In conclusion, we showed that NAC at subinhibitory concentrations, resembling those achievable after systemic or local administration, is able to enhance meropenem and ampicillin/sulbactam activity towards CR-Kp and CR-Ab by inducing a profound alteration of the bacterial cell integrity and, possibly, by interfering with the bacterial redox equilibrium. In future studies, these findings may have important clinical implications for the treatment of MDR-GN infections by exploiting the synergism with beta-lactams and providing further rationale for prospective studies of NAC in patients with MDR-GN infections.

## Figures and Tables

**Table 1:** In vitro activity of different antimicrobials and NAC against carbapenem-resistant (CR) *K. pneumoniae* (CR-Kp, *n* = 15). * Strain #2 was used also for the killing study and for the evaluation of serum bactericidal activity. MEM: meropenem; COL: colistin; TIG: tigecycline; RIF: rifampin; NAC: N-acetyl-cysteine. FICI: fractional inhibitory concentration index (FICI was defined as: ∑FIC: FICA + FICB = MICA + B/MICAalone + MICB + A/MICBalone). Synergism was defined as FICI ≤ 0.5. NA: not applicable.

Strain	MIC NAC (mg/mL)	MIC MEM (μg/mL)	MIC COL (μg/mL)	MIC TIG (μg/mL)	MIC RIF (μg/mL)	NAC + MEM FICI	NAC + COL FICI	NAC + TIG FICI	NAC + RIF FICI
#1	5	256	256	1	16	0.25	>0.5	>0.5	>0.5
#2 *	5	1024	2048	0.5	32	0.5	>0.5	>0.5	0.5
#3	5	16	256	0.5	16	0.25	>0.5	>0.5	>0.5
#4	5	256	256	1	32	0.25	>0.5	>0.5	>0.5
#5	5	256	32	0.5	32	0.37	>0.5	>0.5	>0.5
#6	5	64	1024	0.5	128	0.18	>0.5	>0.5	>0.5
#7	2.5	128	1024	0.5	8	0.37	>0.5	>0.5	0.25
#8	5	256	2	0.25	16	0.25	>0.5	>0.5	>0.5
#9	2.5	512	1	0.25	8	0.5	>0.5	>0.5	>0.5
#10	5	1024	2048	4	1024	0.37	>0.5	>0.5	>0.5
#11	5	1024	8	1	16	0.5	>0.5	>0.5	0.5
#12	2.5	128	16	0.25	16	0.25	>0.5	>0.5	>0.5
#13	5	256	32	0.5	256	0.18	>0.5	>0.5	>0.5
#14	5	512	256	0.5	256	0.25	>0.5	>0.5	>0.5
#15	5	512	64	1	32	0.5	>0.5	>0.5	>0.5
MIC50/90	5/5	256/1024	256/1024	0.5/1	32/256	NA	NA	NA	NA
Synergism, *n* (%)	NA	NA	NA	NA	NA	15 (100)	0 (0)	0 (0)	3 (20)

**Table 2:** In vitro activity of different antimicrobials and NAC against carbapenem-resistant (CR) *A. baumannii* (CR-Ab, *n* = 15). * Strain #8 was used also for the killing study and for the evaluation of the serum bactericidal activity. ° The reported values corresponded to the sulbactam component. MEM: meropenem; A/S: ampicillin/sulbactam; COL: colistin; TIG: tigecycline; RIF: rifampin; NAC: N-acetyl-cysteine. FICI: fractional inhibitory concentration index (FICI was defined as: ∑FIC: FICA + FICB = MICA + B/MICAalone + MICB + A/MICBalone). Synergism was defined as FICI ≤ 0.5. NA: not applicable.

Strain	MIC NAC (mg/mL)	MIC MEM (μg/mL)	MIC A/S ° (μg/mL)	MIC COL (μg/mL)	MIC TIG (μg/mL)	MIC RIF (μg/mL)	NAC + MEM FICI	NAC + A/S FICI	NAC + COL FICI	NAC + TIG FICI	NAC + RIF FICI
#1	5	8	16	1	0.25	4	0.5	0.25	>0.5	>0.5	0.5
#2	2.5	64	128	0.25	0.25	4	0.37	0.5	>0.5	>0.5	0.5
#3	2.5	128	16	4	0.5	2	0.25	0.18	>0.5	>0.5	0.25
#4	5	32	16	0.5	0.5	4	0.5	0.25	>0.5	>0.5	>0.5
#5	5	512	32	0.016	1	32	0.25	0.18	>0.5	>0.5	0.5
#6	2.5	256	32	0.25	0.5	4	0.37	0.37	>0.5	>0.5	0.37
#7	2.5	32	16	8	0.5	2	0.18	0.25	>0.5	>0.5	0.25
#8 *	2.5	1024	32	4	1	8	0.5	0.5	>0.5	>0.5	0.5
#9	2.5	128	64	0.25	0.5	2	0.25	0.25	>0.5	>0.5	0.25
#10	5	64	128	0.25	0.125	2	0.37	0.25	>0.5	>0.5	0.18
#11	2.5	128	64	1	0.5	4	0.25	0.5	>0.5	>0.5	0.5
#12	2.5	128	64	2	0.25	2	0.5	0.5	>0.5	>0.5	0.37
#13	2.5	256	256	0.25	1	8	0.37	0.25	>0.5	>0.5	0.5
#14	2.5	64	64	0.25	0.25	2	0.25	0.5	>0.5	>0.5	0.25
#15	2.5	256	256	0.25	0.5	2	0.18	0.37	>0.5	>0.5	0.25
MIC50/90	2.5/5	128/512	64/256	0.25/4	0.5/1	2/8	NA	NA	NA	NA	NA
Synergism, *n* (%)	NA	NA	NA	NA	NA	NA	15 (100)	15 (100)	0 (0)	0 (0)	14 (93.3)

## Data Availability

Data are available from the corresponding author upon request.
